# The role and value of science in shark conservation advocacy

**DOI:** 10.1038/s41598-021-96020-4

**Published:** 2021-08-17

**Authors:** David S. Shiffman, Catherine C. Macdonald, S. Scott Wallace, Nicholas K. Dulvy

**Affiliations:** 1 Earth to Oceans Group, Department of Biological Sciences, Simon Fraser University, 8888 University Drive, Burnaby, BC V5A 1S6 Canada; 2 Field School Scientific Consulting, Miami, FL USA; 3 David Suzuki Foundation, 2211 West 4th Avenue, Vancouver, BC V6K 4S2 Canada; 4 Present Address: New College of Interdisciplinary Arts and Sciences, Arizona State University, 4701 W Thunderbird Road, Glendale, AZ 85306 USA; 5 Rosenstiel School of Marine and Atmospheric Science, University of Miami, 4600 Rickenbacker Causeway, Miami, FL 33149 USA

**Keywords:** Ichthyology, Environmental impact, Sustainability, Marine biology

## Abstract

Many species of sharks are threatened with extinction, and there has been a longstanding debate in scientific and environmental circles over the most effective and appropriate strategy to conserve and protect them. Should we allow for sustainable fisheries exploitation of species which can withstand fishing pressure, or ban all fisheries for sharks and trade in shark products? In the developing world, exploitation of fisheries resources can be essential to food security and poverty alleviation, and global management efforts are typically focused on sustainably maximizing economic benefits. This approach aligns with traditional fisheries management and the perspectives of most surveyed scientific researchers who study sharks. However, in Europe and North America, sharks are increasingly venerated as wildlife to be preserved irrespective of conservation status, resulting in growing pressure to prohibit exploitation of sharks and trade in shark products. To understand the causes and significance of this divergence in goals, we surveyed 155 shark conservation focused environmental advocates from 78 environmental non-profits, and asked three key questions: (1) where do advocates get scientific information? (2) Does all policy-relevant scientific information reach advocates? and (3) Do advocates work towards the same policy goals identified by scientific researchers? Findings suggest many environmental advocates are aware of key scientific results and use science-based arguments in their advocacy, but a small but vocal subset of advocates report that they never read the scientific literature or speak to scientists. Engagement with science appears to be a key predictor of whether advocates support sustainable management of shark fisheries or bans on shark fishing and trade in shark products. Conservation is a normative discipline, and this analysis more clearly articulates two distinct perspectives in shark conservation. Most advocates support the same evidence-based policies as academic and government scientists, while a smaller percentage are driven more by moral and ethical beliefs and may not find scientific research relevant or persuasive. We also find possible evidence that a small group of non-profits may be misrepresenting the state of the science while claiming to use science-based arguments, a concern that has been raised by surveyed scientists about the environmental community. This analysis suggests possible alternative avenues for engaging diverse stakeholders in productive discussions about shark conservation.

## Introduction

The most effective and appropriate way to conserve and manage the environment has long been debated^1^, exemplified in the United States by the rational utilization approach of Gifford Pinchot (i.e., active management of resources with the goal of exploiting them with minimal impacts on the environment) versus the preservationist approach of John Muir (i.e., fully protect areas of pristine wilderness and associated wildlife). There is broad public agreement about which of these approaches to apply to some environmental problems; few Americans would dispute that the great whales are “wildlife” to be preserved from exploitation while anchovies are “natural resources” to be sustainably exploited (for more on the framing and language around these issues, see^2^). Conflicts within the environmental community, and between the environmental community and other stakeholder groups, can occur when there is a dispute over which philosophical approach applies to specific environmental issues^3^. The debate over the best approach to take when conserving and managing chondrichthyan fishes (sharks and their relatives, though in this paper we focus exclusively on sharks) is an interesting case study of such a conflict. Some stakeholders assert that sharks are natural resources to be sustainably exploited, and others assert that sharks are wildlife to be preserved.

Chondrichthyan fishes (sharks, rays, skates and chimaeras) comprise over 1200 species, and include some of the most threatened vertebrates on Earth, due largely to overfishing^4^. However, one quarter of species are considered Least Concern by the IUCN Red List^4^, and many are sustainably exploited^5^. Since sharks can be ecologically important [e.g.,^6^] and are a popular encounter for scubadivers and other marine tourists [e.g.,^7^], issues surrounding their conservation have resulted in significant public interest and concern^8^.

Possible conservation solutions for threatened sharks can be broadly categorized into two main policy families: “target-based” solutions (e.g., traditional fisheries management) which are intended to maximize sustainable fisheries exploitation, and “limit-based” solutions (e.g., bans on shark fishing within a country’s entire EEZ termed “Shark Sanctuaries,” or bans on the sale of shark fins), which are intended to ban all fisheries exploitation and trade of all sharks regardless of the sustainability of a particular fishery or the health of a particular stock^9^. While conservationists often generally agree on broad principles and goals^10^, mutually exclusive goals (i.e., a nation cannot simultaneously promote sustainable fisheries while banning all fishing at least in the same place and time) can create disagreement or even conflict between different environmental groups or between stakeholders^11^. While a country’s management strategy can certainly contain a mixture of target-based policies and limit-based policies (e.g., sustainable fisheries exploitation for species whose populations can withstand fishing pressure and no-take marine protected areas,) it is notable that some limit-based policies are intended to be nationwide in nature and preclude the inclusion of target-based policies into a management framework. Generally, the goal of most industrialized nations’ fisheries management strategies is sustainable fisheries management for species that can withstand fishing pressure, and this has been true for a long time^9^.

Support for limit-based policies is sometimes tied to a belief that sustainable shark fisheries cannot or do not exist^12^ and that bans are therefore the only possible solution. Support for target-based policies is likely to correlate with beliefs that sustainable shark fisheries can and do exist, and offer social, economic, or conservation benefits not available under limit-based bans, including contributions to food security and local livelihoods, as well as fisheries-dependent data-gathering. The scientific literature is clear that shark fisheries can^13^ and do^5,14^ exist, though the majority of the world’s shark fisheries are currently unsustainable and many likely cannot be made sustainable under current management regimes. While proponents of limit-based policies often point to genuine evidence-based concerns about the practicality of implementing sustainable fisheries for sharks, some limit-based proponents sometimes appear to misunderstand or misrepresent the science^9,11^.

Conservation and management planning has long been dependent on expert opinions, especially those of professional scientists [see^15^ for how this system works for shark management in the United States]. A recent survey of members of the American Elasmobranch Society, the oldest and largest professional society focusing on the scientific study and management of chondricthyan fishes, found that majorities of surveyed scientists agree that sustainable shark fisheries are possible (84%), currently exist in the world today (83%,) and should be the goal of conservation advocacy over bans when possible (90%)^11^. These AES scientists also showed significantly greater support for target-based policies versus limit-based policies. Additionally, surveyed experts expressed concern that some environmental non-profit groups working in the shark conservation space were focusing on problems that the data showed were not the most critical problems, advocating for ineffective solutions, and/or in some cases miscommunicating or misunderstanding information related to shark conservation threats and policy solutions^11^. While values-based approaches to conservation have contributed significantly to numerous successful advocacy campaigns (e.g., early anti-shark-finning advocacy focused on both the sustainability concerns and the animal welfare concerns bringing together multiple stakeholder groups), issues can arise when proponents of such an approach use misleading or inaccurate appeals to scientific authority to support their values-based advocacy^16^.

This paper represents a case study on sharks which illuminates philosophical divisions over exploitation versus preservation more broadly, and discusses how these divisions have contributed to difficulties in communication around conservation issues between scientific experts and some members of the shark conservation advocacy community. Here we report on the results of a survey sent to employees of environmental non-profit organizations who work on shark conservation advocacy and public education. The survey focused on three general questions: (1) Where do environmental advocates get their scientific information? (2) Does all relevant scientific information reach environmental advocates? and (3) Do environmental advocates report working towards the stated policy preferences of scientists?

## Methods

### Identification of shark conservation NGO employees

We identified 78 environmental non-profits that participate in shark conservation advocacy or public education in the English-speaking world (Supplementary materials Table S1) using a combination of internet search engine searches for shark conservation advocacy, our own records from combined decades working on ocean conservation science issues, and snowball sampling (i.e., asking contacted advocates to suggest other organizations we should be sure to include).

Representatives of each non-profit were contacted via e-mail and asked to provide a list of contact information for anyone at their organization or partner organizations who works directly on these issues. We compiled a base list of 155 names of employees of environmental non-profit groups whose job focuses on shark conservation advocacy and/or public education (henceforth “environmental advocates”). Shark conservation advocacy and public education was defined broadly to capture as much of the diversity of thought and action in this space as possible, but did not include employees of non-profit groups whose primary duties included scientific research, as the intended focus of this study is individuals engaged in advocacy, outreach, and education.

#### Survey

These 155 individual environmental advocates were sent an individualized but anonymized link to participate in a voluntary online survey hosted through SurveyMonkey.com. The survey included 49 questions, focusing on environmental advocates’ perspectives on shark conservation policies and relevant science (Supplementary Materials Table S2). Respondents were promised no compensation for completing the survey and were permitted to stop answering questions at any time.

We included questions relating to demographic background (age, gender, education), NGO background (size, scope, role in the advocacy process), science knowledge and attitudes (questions concerning advocates’ past experience working with scientists, their perspectives on the state of scientific research, and how well-versed they are in the current scientific literature) conservation background questions (thoughts on threats facing sharks and the general issues surrounding shark conservation), and policy preferences (support for and opposition to certain specific shark conservation policies).We also asked about awareness of rebuttals (scientific papers disputing the results of previous scientific papers), which were used as a proxy for awareness of technical information related to shark conservation and management. All respondents were offered the opportunity to participate in 30-min follow-up interviews over Skype, and eight respondents chose to participate in a follow-up interview. These respondents included representatives from four countries, employed by large ocean focused non-profits as well as small regional shark-focused non-profits. Responses from follow-up interviews were used only to provide representative examples of certain attitudes and no statistical analysis was performed on these results.

Average completion time of the survey was 43 min. Respondents who did not complete the entire survey had the answers they did provide analyzed, and the blank responses were not counted. This research is approved under Simon Fraser University’s Office of Research Ethics permit # 2017-S0524. All research was performed in accordance with all relevant human subjects research ethics guidelines and regulations, and as explicitly stated in the recruitment materials, participation in the survey constituted informed consent.

### Statistical analysis

Analyses focused on determining patterns in support of or in opposition to certain conservation policies, as well as patterns in awareness of scientific background information. Variables analyzed included demographic background of the respondent and information about their employer (e.g., are people that work for large non-profits with easy access to scientific expertise more likely to support the policy goals identified by scientists than employees of smaller non-profits without easy access to scientific expertise, etc.) All statistical analysis was performed using R software (R version 3.5.2 2018-12-20—"Eggshell Igloo").

We used conditional inference trees to determine the primary partitioning variables (demographic or NGO background) responsible for particular outcomes (awareness of scientific research and support for certain policies) using the PartyKit package in R^17^. These trees highlight which variable is responsible for the greatest difference in output by fitting models to each combination of variables, and splitting the dataset at the variable associated with the greatest divergence in output. Conditional inference trees were run to determine the primary partitioning variables associated with the following outputs: general preference for sustainable fisheries or for total bans, support for or opposition to shark fin trade bans, awareness that sustainable shark fisheries exist, and technical understanding of the state of published scientific evidence concerning sustainable shark fisheries.

### Demographics of survey respondents

More than half of contacted environmental advocates completed the survey (54.2% response rate, N = 84). However, many questions were optional and only applicable for respondents who had worked on particular issues. The response rate on optional questions ranged from 26.2 to 67.8% While this is a low N overall, we believe that it represents a major segments of the entire English-speaking world’s population of shark conservation advocates and educators, as our method for identifying non-profits to approach for this survey included extensive personal records, search engine searches, and the opportunity for non-profits we contacted to suggest collaborators working for other groups. However, this approach may have missed smaller regional non-profits outside of North America and Europe who do not have a large online presence and have not worked extensively with international collaborators. Fifty-seven percent of respondents identified as female, and one respondent preferred not to answer this question. Respondents ranged in age from 24 to 70, with a mean age of 41 years old. Age or gender were not significantly correlated with any variables measuring understanding of current science or support for any management policy and were not analyzed further. Thirty-seven respondents have a Master’s degree, seventeen have a Ph.D, and the remainder have undergraduate degrees or did not answer this question.

**Figure 1:**
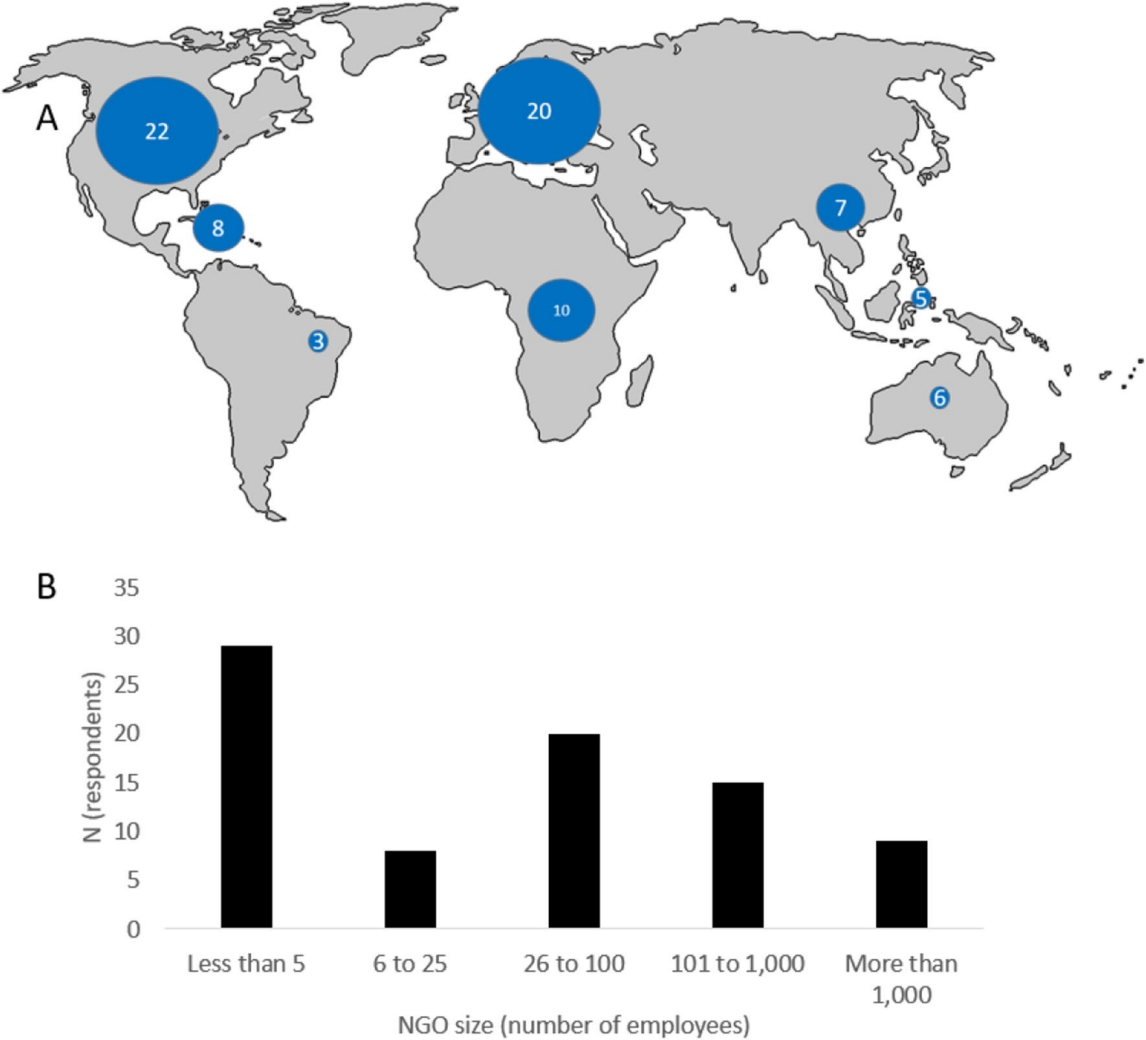
(**A**) Where respondents primarily work, excluding those who responded with “worldwide,” with bubbles scaled using PowerPoint (**B**) distribution of the size of non-profits (by number of employees) that respondents self-report working for.

Non-profits were classified by scope (shark-focused, ocean-focused including but not limited to sharks, or focused on all environmental issues including but not limited to sharks), area of geographic focus, and size (number of employees). Forty-six percent of respondents worked for an ocean-focused conservation NGO that included a shark portfolio (e.g., Oceana), while thirty-two percent worked for an exclusively shark-focused NGO (e.g., the Shark Trust). The remaining twenty-two percent of respondents worked for large NGOs that focus on a variety of land and sea conservation issues (e.g., World Wildlife Fund). Of those who did not claim that the scope of their work was global, the largest proportion of respondents worked in North America and Europe, followed by the Caribbean, South Africa, and Southeast Asia. Some respondents also worked in Central America, Brazil, the South Pacific, and Australia (Fig. 1A).

Respondents reported that the size of their non-profit ranged from zero paid employees (i.e., all volunteer) to over 5000, with a median of 14 employees (Fig. 1B). Twenty-four respondents reported working for a non-profit with more than 100 employees, 36 reported working for a non-profit with fewer than 5 employees.

## Results

### Where do environmental advocates get their scientific information?

Two-thirds of respondents indicated that they regularly read several peer-reviewed primary scientific literature articles each month, and only eight percent of respondents indicated that they had never read a peer-reviewed primary literature article. All respondents who reported having never read the literature worked for a non-profit with fewer than 10 employees. Fifty-five percent of respondents indicated that they had been a coauthor or lead author on at least one peer-reviewed primary scientific literature article.

Fifty-six percent of respondents reported that scientists were directly employed by their non-profit, and 16% reported that their non-profit had a formal scientific advisory board composed of independent scientists who were available for technical consultation. Only 12% of respondents indicated that their NGO did not work with any scientists in any capacity (each of the 8% of respondents who reported never having read a scientific article also reported never working with scientists in any capacity). Of the respondents who did not work with scientists in any capacity, 75% reported working for very small (less than five employees) shark-focused NGOs in Europe and North America.

**Figure 2:**
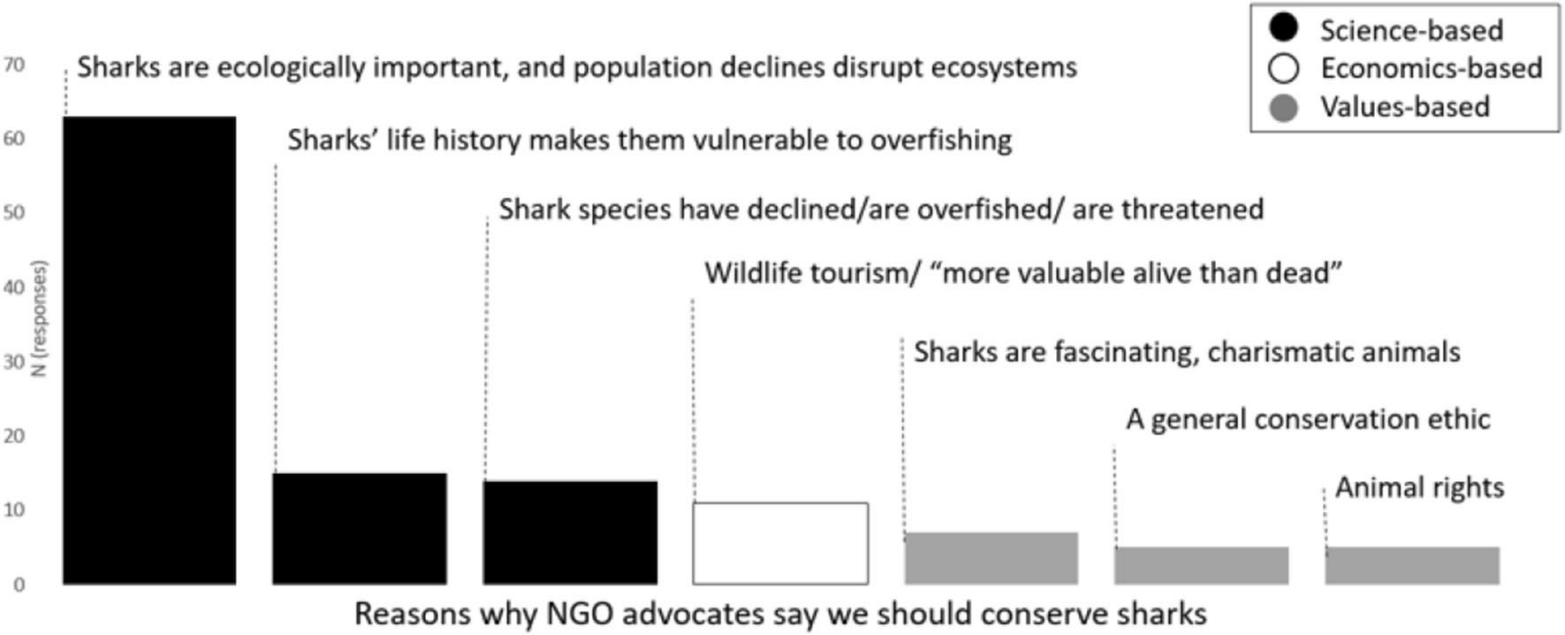
Categorized responses to the question “What arguments does your NGO make to conserve sharks,” with science-based, economics-based, or emotional arguments as possible categories.

Respondents reported that science-based arguments were by far the most commonly advanced arguments for shark conservation used by their NGO (Fig. 2, Supplementary Materials Table S3), especially the idea that shark population declines can cause negative ecosystem-wide effects. Respondents reported that moral or values-based arguments were much less frequently employed than science-based arguments.

### Does all relevant scientific information reach environmental advocates?

Surveyed environmental advocates were most aware of scientific papers showing severe shark population declines and papers showing negative ecological consequences of those declines (97% and 100% awareness respectively). While rebuttals to those papers (72.7% awareness) and papers showing that that sustainable shark fisheries are possible (82.6% awareness) were still widely known, they had lower awareness among respondents compared with papers showing population declines and ecological consequences of those declines. Some widely-publicized papers about shark conservation have been considered somewhat controversial by other scientists in the field resulting in rebuttals. Rebuttals can be seen as an attempt to correct the scientific record by pointing out an error in the first paper, therefore awareness of the rebuttals was considered to be a proxy for technical knowledge related to the current state of scientific evidence of shark conservation and management issues.

In follow-up interviews, respondents reported actively looking for rebuttals whenever they found a paper that appeared to support their perspective. One explained “*We look for it all, and we’re always open to using new science that comes along and tells us something different.*” Another said, “*We always try and include rebuttals and contradictory data to provide the whole picture.*” A third told interviewers that “*It’s too easy to see a paper that justifies your claim that you can jump on and use, but how valid is that?*” Interviewees consistently stressed the importance of seeking out data that not only supported their arguments but was scientifically valid.

Some of the environmental non-profit advocates surveyed here also raised concerns about the focus of most shark research produced by academic scientists. Several respondents suggested that research could be made more useful by broadening the current focus (a few well-studied species in a few well-studied regions) to include less charismatic species and less-visited study sites, especially those in the developing world. Respondents also raised concerns about scientists claiming to do conservation-relevant research without consulting managers, local colleagues, or members of the impacted community to see what kind of data would be most useful. One follow-up Skype interview participant said that if scientists want to do policy-relevant research, “*The first step is to try and identify information needs of policymakers; they know what they don’t know and what they need to know- talk to them as early as possible when starting a research project!*”.

Respondents also suggested new roles for scientists, such as serving as public educators or advocates for conservation by communicating their research to the public. Several respondents from the developing world suggested the scientists from wealthy institutions or nations should cease engaging in “helicopter science” or “parachute science”^18^ by visiting faraway places and leaving as soon as their research was done (while this term can also refer to exploitative relationships with geographically proximate indigenous communities, we note that our respondents were clear that they meant international applications of this termInstead, respondents request that visiting scholars provide training and opportunities to colleagues and students in the developing world to develop local capacity. There were also calls for more research on the human dimensions of shark conservation, including socioeconomic studies of shark fishers.

### Do environmental advocates work towards the stated policy preferences of scientist?

**Figure 3:**
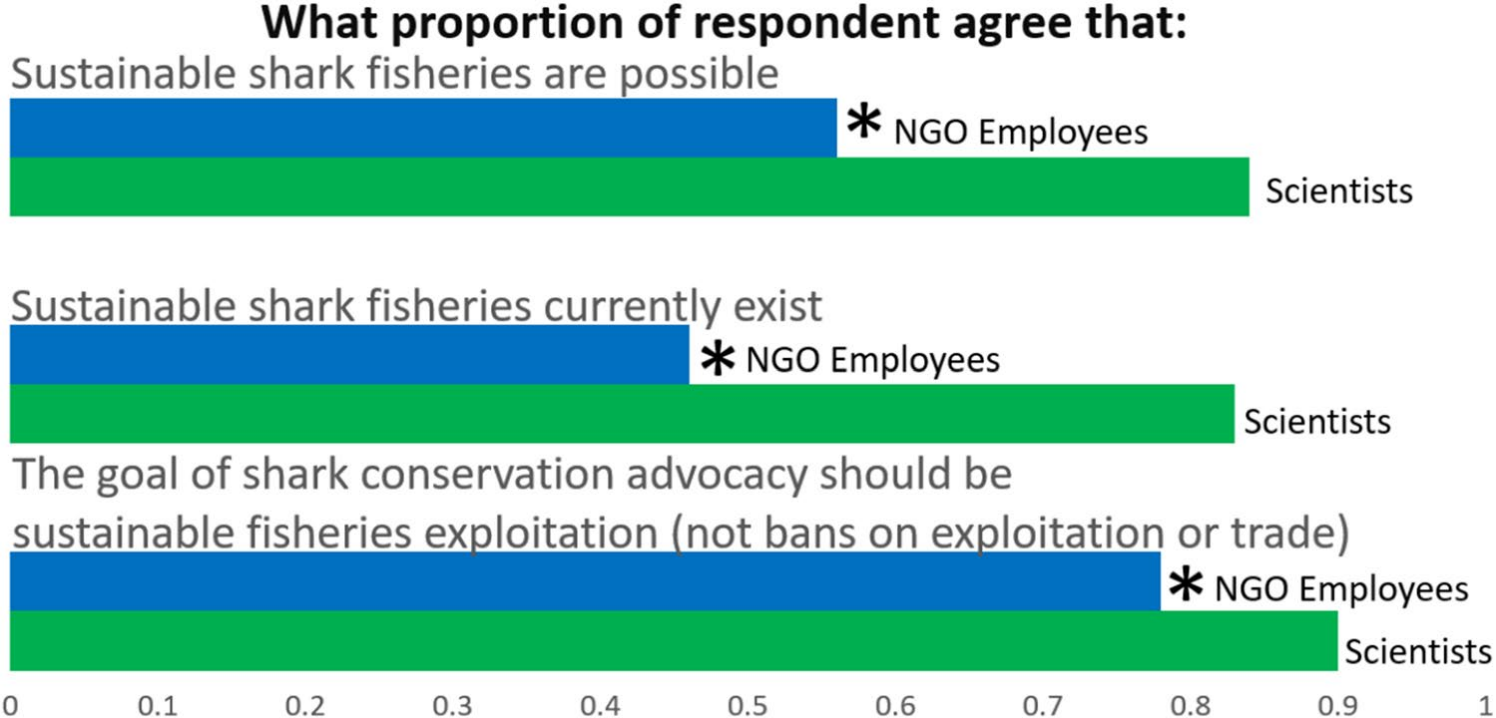
Proportion of environmental advocate respondents from this study (blue) and scientists respondents from Shiffman and Hammerschlag 2016B (green) who agree with these three questions, with * indicating a significant difference in responses to each question between the two groups using a 2-tailed Z test of independent proportions (for Question 1, Z =  − 3.66, *P* = 0.0003, for Question 2, Z =  − 4.48, *P* < 0.00001, for Question 3, Z =  − 1.87, *P* = 0.03).

Over half (56%) respondents correctly identified that published scientific evidence shows that sustainable shark fisheries are possible, and nearly half (46%) correctly identified that published scientific evidence shows that sustainable shark fisheries exist in the world today (there is no factual or scientific doubt that such fisheries can and do exist; while preferring bans based on personal values is a valid approach, claiming that bans are universally necessary because sustainable fisheries are scientifically impossible is a misrepresentation of the science). More than three-quarters (78%) of respondents agreed with the statement that the goal of shark conservation advocacy should be to promote sustainable exploitation instead of complete bans on exploitation and trade (Fig. 3, Supplementary Materials Table S4). In each case, significantly fewer NGO employees than previously-surveyed AES scientists agreed with these statements.

**Figure 4:**
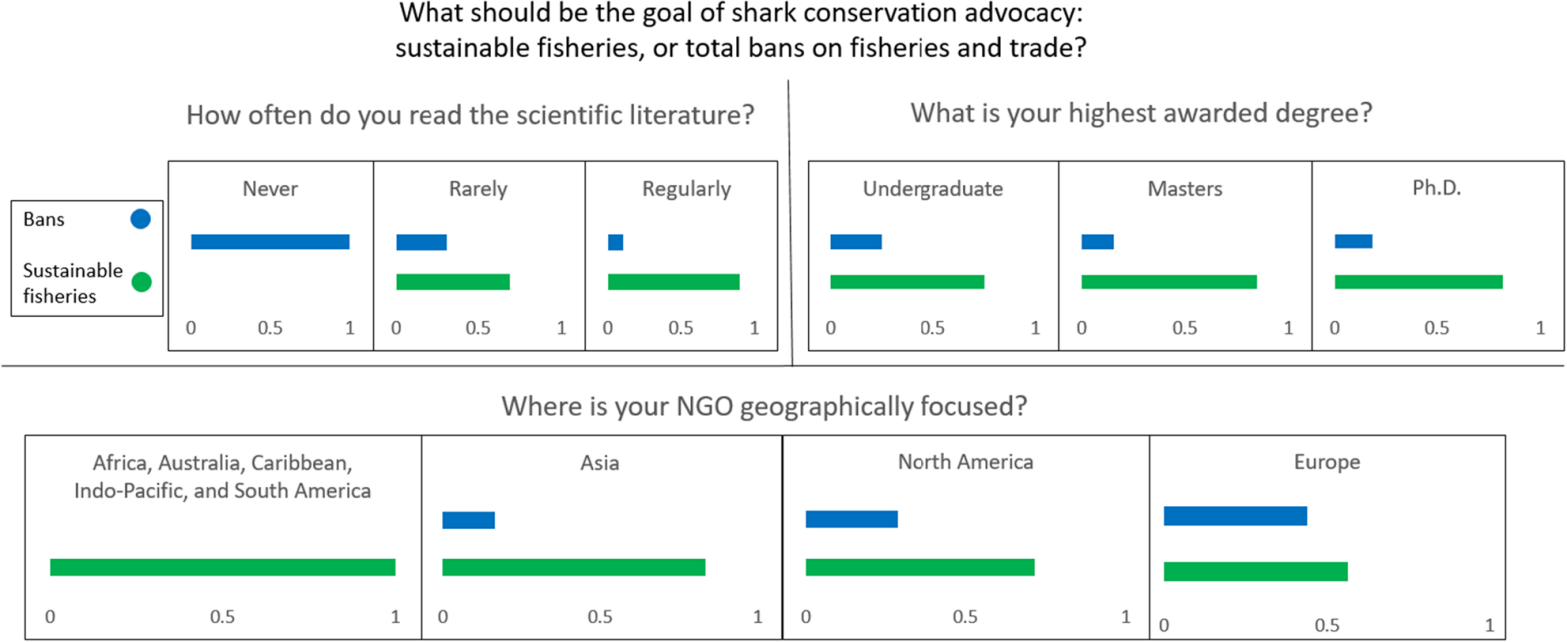
Proportion of environmental advocate respondents who agree with the statement that sustainable fisheries (as compared with bans on all fishing and trade) should be the goal of shark conservation, broken down by regularity of reading the literature, highest degree earned, and geographic area of focus.

**Figure 5:**
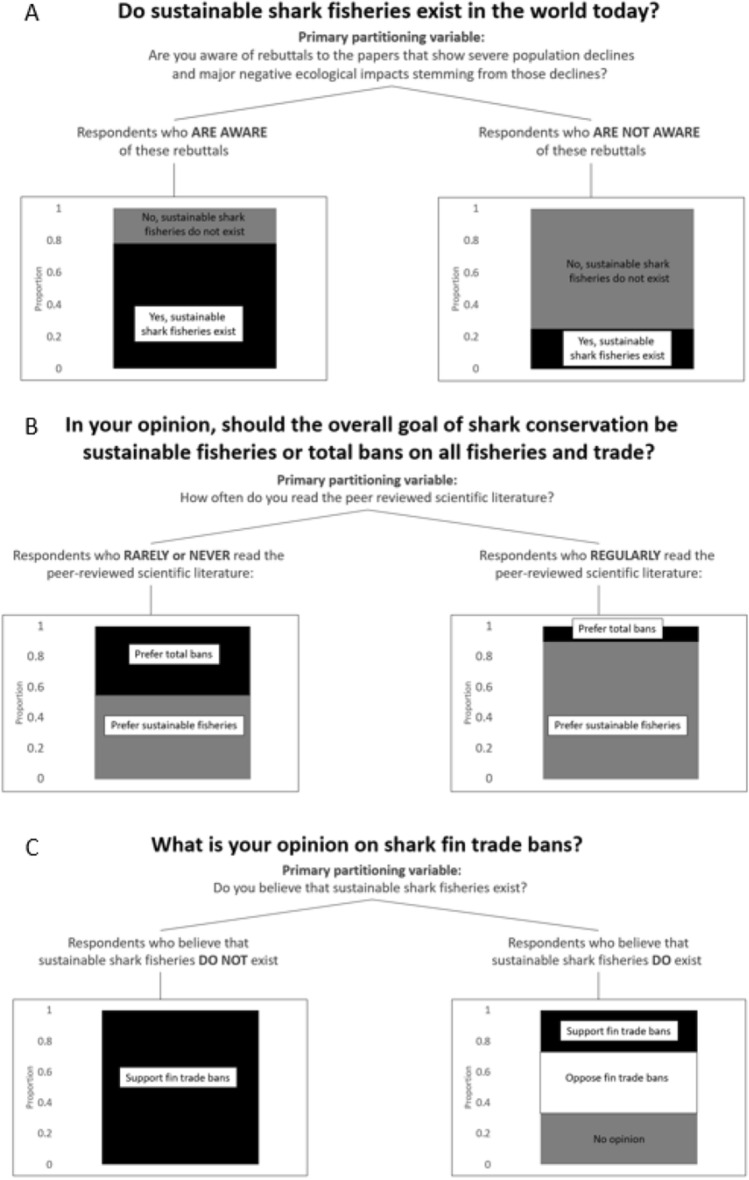
Results of conditional inference trees for the questions “should the goal of shark conservation be sustainable fisheries or total bans on fisheries and trade,” “do you support shark fin trade bans,” and “do sustainable shark fisheries exist in the world today,” showing primary partitioning variables for each and the results of separating the data by those partitioning variables.

Results show that in general, the environmental advocates who most strongly supported bans on fisheries and trade were the least familiar with the current state of scientific knowledge on sustainable shark fisheries. While many of these respondents reported that science was important to advocacy and that their arguments were based on science, many arguments misrepresented the state of the science. A conditional inference tree found that the primary partitioning variable associated with general support for bans on trade was self-reported regularity of reading the scientific literature; 100% of respondents who report never reading the scientific literature (N = 4 of 4) supported bans over sustainable fisheries, compared with just 10.5% of respondents who reported regularly reading the scientific literature (N = 4 of 38), (Figs. 4, 5). There was also a clear geographic bias with respondents who worked in the developed world more likely to support bans than those working in the developing world (Fig. 4).

Of respondents who supported a total ban on all exploitation and trade over sustainable fisheries, 86% work in the US or Europe, while 100% of respondents who work in South America (N = 3), the Indo Pacific (N = 1), the Caribbean (N = 1), or Africa (N = 1) who answered this question preferred sustainable fishing over bans (Fig. 4). One-third (N = 7) of respondents who work at shark-specific environmental non-profits support bans over sustainable fisheries, compared to just 12% (N = 3) of those who work in ocean-focused environmental non-profits. Fifty percent of all stated preferences for bans over sustainable fisheries came from advocates working for very small (less than five employees) non-profits.

Respondents who did not agree that sustainable shark fisheries are possible or exist cited a variety of reasons, ranging from not having personally seen evidence of sustainability to technical concerns to a general overarching belief that sustainable fisheries in general cannot and do not exist (Table 1).

**Table 1:** Representative example responses showing reasons why respondents do not agree with the statement that sustainable shark fishing is possible, including references to literature on this topic.

I have never seen a truly sustainable shark fishery and have never seen evidence that it would be possible to maintain
Because of some of the "misses" that we have here in the US, in that we don't have stock assessments for most of the species that are commercially fished so there are still a lot of unknowns
I have read some for and some against on this point. Much like MPA literature, it seems a very polarized place
I have not come across any studies that show true sustainability
Sustainable shark fisheries will always be at the mercy of the unscrupulous who will ignore the rules and seek to evade enforcement issues in order to make a quick, short-term profit
Several shark fisheries have not been evaluated
Data is lacking to understand the health of shark populations. Sharks are often part of a complex multi-species fishery and caught as bycatch creating challenges for data collection and assessment. Due to the high vulnerability of many species to overfishing it is necessary to take precautionary measures to reduce fishing pressure while data is improved
"Sustainability" is over-used politically and an illusion in many ways. It sounds nice in political documents but I do not think that governments yet understand the true meaning nor have any idea how to realize it, not just for sharks
Many species lifespans are time until sexual maturity is too long to allow them to sustain fishing
No sustainable shark fishing is possible!!!
I think there is a HUGE gap between the scientists who study fisheries in well-resourced countries with good fisheries governance structures, and marine scientists (of all sorts, not just fisheries folk) who have seen what "fisheries management" looks like in the rest of the world

The individual who reported never having personally seen any evidence of sustainable shark fisheries also reported never having read the scientific literature and never interacting with professional scientists. Those respondents who did agree that sustainable shark fisheries are possible and exist mostly cited peer-reviewed published technical literature to support their opinion (Table 2), though several noted that while these fisheries can and do exist, there are not very many of them, and not all shark fisheries are potentially sustainable.

**Table 2:** Representative example responses showing reasons why respondents agree that sustainable shark fisheries are possible and exist.

I have read papers that show that certain shark fisheries can be sustainable and some currently are
More research needs to be done I believe, but there is sufficient science to suggest these fisheries can and do exist
I am engaged in fisheries management, keep up on status reports, and find that scientists usually present facts
Yes, sustainable shark fisheries are possible, but currently they are very rare and have significant weaknesses
It is clear that they can and do exist but this does not mean that all shark fisheries are or even could realistically be sustainable
Sharks are only viewed as a special species by a small minority of nationalities, usually ones that do not have any food security issues. For many vulnerable coastal communities, sharks are just a different food resource
The misconceptions around shark fisheries fueled by extreme NGOs is damaging. We need to work hard to educate the public on sustainable fisheries not push for the sensation stories. We also need NGO's to back up their stats with science. We do this—others should to

This suggests that while some advocates are not aware of the current state of science on this topic or are misinformed about it, for some, the issue is less about knowledge of whether sustainable shark fisheries are possible in theory than about whether they personally believed successful implementation of sustainable management practices was probable or practical in the complex real world—there are certainly many examples of poorly managed shark fisheries, a point broadly understood both by those advocating for improving fisheries sustainability and those advocating for bans.

One respondent explicitly mentioned misinformation from other non-profits as a possible cause of public misunderstanding on the issue of sustainable shark fisheries. A conditional inference tree indicated that the primary partitioning variable driving agreement with the statement that sustainable shark fisheries exist was awareness of rebuttals to high-profile shark conservation papers, which was used as a proxy for awareness of the current state of technical literature (Fig. 5). Only 24% of respondents who were unaware of those rebuttals agreed that sustainable shark fisheries exist, compared to 78% of those who were aware of these rebuttals. Of respondents who report regularly reading the scientific literature, 88.6% (N = 39) agree that there are current examples of sustainable shark fisheries, compared with just 50% (N = 2) of those who never read the literature and 62% (N = 10) of those who rarely read the literature. Sixty-five percent of all respondents who agree that sustainable fisheries do not exist come from the US or Europe, and no respondents in the Caribbean, South America, or Africa agreed with the statement that sustainable shark fisheries do not exist.

A plurality of respondents (45.8%) agreed with the statement that the science concerning the sustainability of shark fisheries is currently uncertain, and 15.2% of respondents agreed with the statement that the science is clear that sustainable shark fisheries cannot and do not exist. Sixty-five percent of respondents with a Masters or Ph.D. degree correctly identified that the science is clear that sustainable shark fisheries can and do exist (and 0 respondents with a Masters of Ph.D. degree indicated that the science is clear that sustainable shark fisheries cannot and do not exist). There was also a divide by familiarity with the scientific literature, none of the respondents who never read scientific papers correctly identified that the science is clear that sustainable shark fisheries can and do exist, compared with 26.6% of those who rarely read the literature and 45.2% of those who regularly read the literature. Fifty percent of respondents who never read the literature inaccurately reported that they agree that the science is clear that sustainable shark fisheries cannot and do not exist, compared with 26.6% of those who rarely read the literature and 9.5% of those who regularly read the literature. Nine percent of respondents reported that their opinions about shark fisheries come from their personal ethical values, and therefore scientific measures of sustainability are not relevant to their decision-making on this topic.

**Figure 6:**
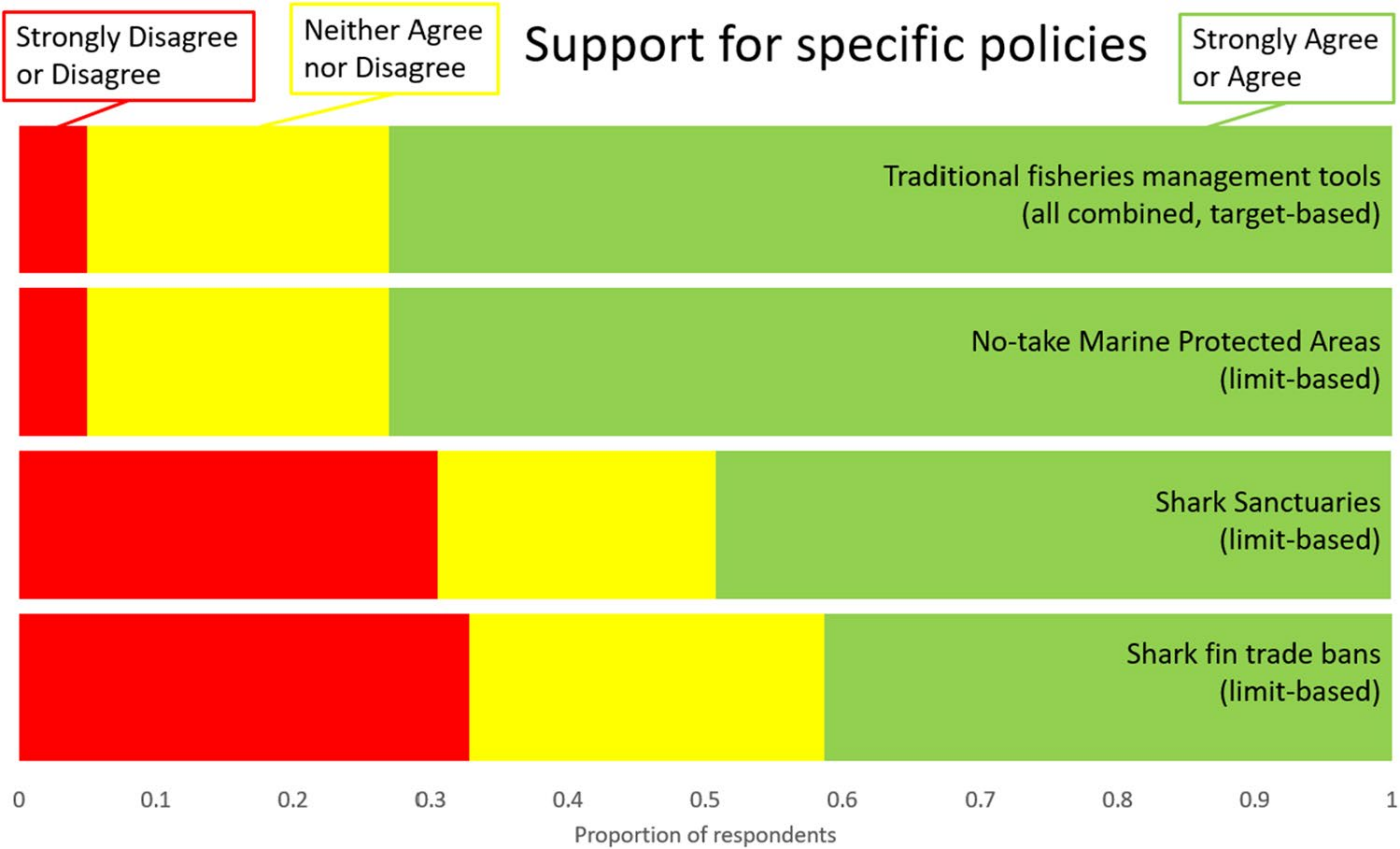
Proportion of respondents who support, oppose, or have no opinion concerning a variety of conservation policies. All target-based traditional fisheries management tools were grouped together because there were no differences in responses.

Fifty-four percent of respondents reported that their environmental non-profit organization has worked on fisheries management tools in the last five years, compared with lower numbers of those whose employer worked on no-take marine protected areas (45.2%), fin bans (25%), and Shark Sanctuaries (16.7%). Many respondents noted that different contexts (cultural, political, and economic) require different kinds of solutions, that there is no one “silver bullet” policy for shark conservation, and that enforcement of existing management rules is critical no matter which policy strategy is selected. Significantly more respondents support traditional fisheries management tools (73.3%) than support either Shark Sanctuaries (49.1%) or shark fin trade bans (41%), and significantly more respondents oppose Shark Sanctuaries (30.1%) and shark fin trade bans (32.7%) than oppose traditional fisheries management tools (5%) (Fig. 6). There was no difference in support for or opposition to no-take marine protected areas versus traditional fisheries management tools. The only respondent who strongly disagreed with traditional science-based fisheries management tools also reported never reading the literature or interacting with a scientist.

**Figure 7:**
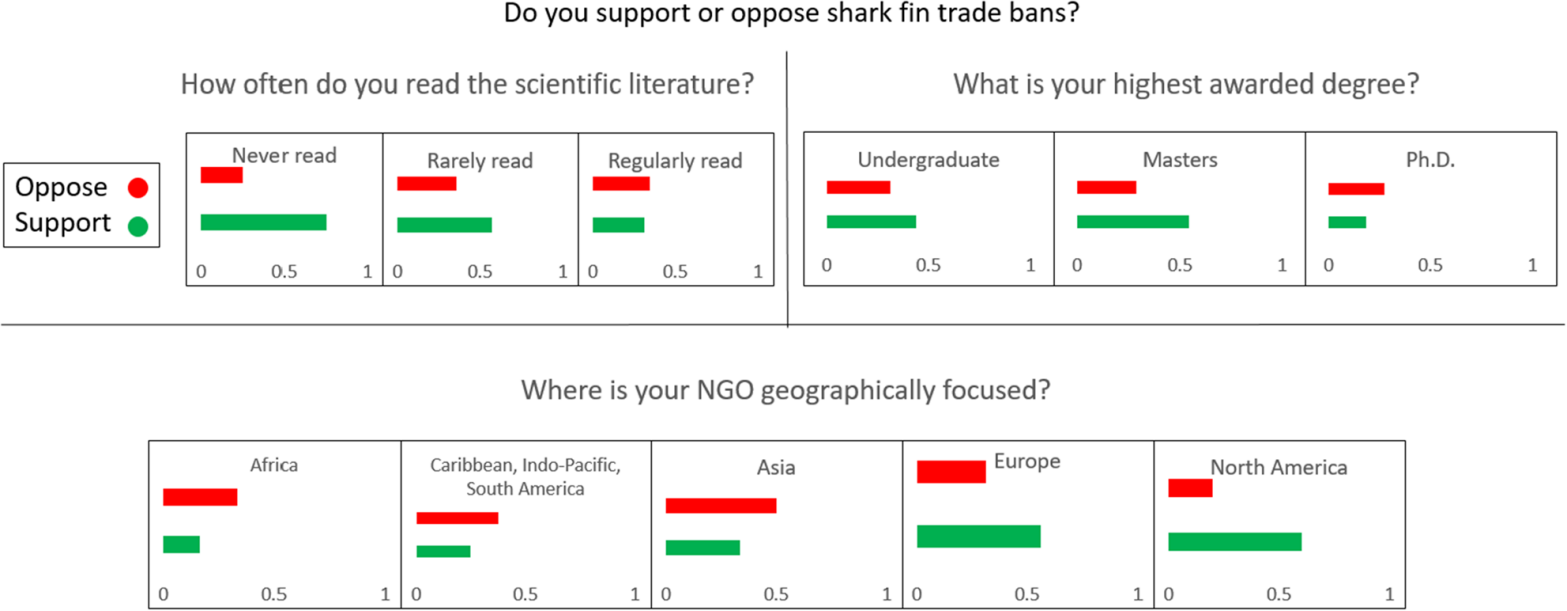
Proportion of respondents who support or oppose shark fin bans (neutral responses/no opinion removed), broken down by regularity of reading the literature, highest degree earned, and geographic area of focus.

A conditional inference tree indicated that the primary partitioning variable associated with support for shark fin trade bans was agreement with the statement that sustainable shark fisheries cannot exist (Fig. 5); 100% of respondents who agree that sustainable shark fisheries cannot exist support shark fin trade bans, compared with 24% of respondents who agree that sustainable shark fisheries can exist. Respondents with a Ph.D. showed the least support for shark fin bans (18.1%, compared to 43.7% support from respondents with a Bachelor’s degree and 54.1% support from respondents with a Masters). Respondents who regularly read the literature had the lowest support for fin bans (31.5% support, compared with 57.1% support from those who rarely read the literature and 75% support from those who never read the literature) (Fig. 7). The only two geographic regions where more respondents supported fin bans than opposed them were Europe (56.2% support) and North America (60% support). In Asia, 50% of respondents opposed fin bans compared to 33.3% who support them. No respondents from the Caribbean supported fin bans. The Indo-Pacific and South America had equal numbers of supporters and opponents.

## Discussion

We reveal here some of the possible drivers of poor communication and misunderstanding between stakeholder groups concerned about shark conservation. Our results provide important context about factors shaping policy preferences for conservation groups, showing further evidence of a target-based versus limit-based schism in shark conservation advocacy. We also provide information that may help explain how that schism formed, and potential strategies for improving communications.

Many environmental advocates in the shark conservation space work for scientifically informed and scientifically engaged non-profits—these environmental advocates regularly read the scientific literature, regularly engage with scientists, and work towards some of the policy preferences supported by scientists. This suggests that science can indeed influence and assist with advocacy and policy change, heartening information for scientists who want their research to make a difference^19^.

However, a number of smaller shark conservation non-profits are not scientifically informed, even as some of these non-profits claim to base their arguments on scientific facts. Employees of these non-profits were not aware of relevant scientific information, and in some cases mischaracterized relevant scientific information while supporting policies that are the least supported (and most opposed) by surveyed scientists. There is no doubt that conservation is a normative discipline and values can play an important role; animal welfare concerns rather than sustainability concerns drove some of the earliest restrictions on shark finning, for example. However, ignoring relevant science can lead to suboptimal policy outcomes^16^, and evidence suggests that focusing only on the perceived cruelty of shark finning and not on the unsustainability of associated overfishing of sharks did nothing to reduce overall shark mortality in the Pacific Ocean^20^. Environmental advocacy based on moral or ethical beliefs is entirely valid and can play an important role in driving policy change, but interviewees repeatedly noted that it is problematic to offer false or misleading information as part of “science-based” arguments for conservation. In other words, approaching conservation discussions with arguments that do not involve scientific facts is a valid and important approach, but when claiming to use scientific facts it is important that those facts be accurate.

It is also worth noting that many of the concerns about the current focus of scientific research raised by surveyed environmental advocates are legitimate, and any scientist concerned about misrepresentation of science by advocates should also be concerned with improving the quality and diversity of conservation-relevant scientific data. Calls for scientists to focus our efforts on species that have not already been well-studied or on places where there is little scientific infrastructure in place (and to develop local capacity while doing so) point to a genuine problem with the state of shark research. Requests that scientists learn how policymaking works so they can generate more policy relevant research illustrates one way that science could provide greater value to managers. Increased engagement with the public is another worthy avenue to pursue, especially in a field where many scientific experts hope to use their data to improve management and conservation outcomes^11,21^.

While many advocates report regularly reading the literature, not all literature is equally well-known. Research showing severe population declines and the negative ecosystem consequences of those declines is better known than rebuttals to those papers, supporting the assertion by^22^ that rebuttals have value, but may not substantially adjust future usage of a rebutted paper. It should also be noted that smaller non-profits who do not read the scientific literature may simply not have the resources to afford access to scientific journals and databases, which can be quite expensive [see^23^]. Though several employees of non-profits surveyed here explicitly stated that this was not the case for them, it is possible that easier, more affordable access to published literature and expert scientific advice for smaller NGOs could help address some of this divide.

Advocates who regularly read the literature, advocates based in the developing world, and advocates with advanced degrees supported sustainable fisheries more than those who never or rarely read the literature, those in North America and Europe, and those without advanced degrees, respectively. While it is important to note that skeptics of sustainable shark fisheries management raised valid concerns regarding the historical unsustainability of shark fisheries and difficulties with implementing sustainable management in some nations, it is demonstrably true that sustainable shark fisheries can and do exist^5^, and claims to the contrary misrepresent the state of scientific knowledge. It is also noteworthy that supporters of the goal of sustainable fisheries are more likely to come from the developing world where such fisheries are relatively rare, and supporters of total bans are more likely to come from developed nations where successful sustainable fisheries management is more common. This may be because a ban on fishing is less feasible in less prosperous nations where fishing is an economically critical activity vital to local food security. Additionally, some types of animal welfare concerns (e.g., animal cruelty in food production) may be more common in developed nations.

While a majority of advocates work towards target-based fisheries management policies, some work towards the policies least supported by scientists. Interestingly, while of course some shark fin trade ban proponents are scientifically informed and engaged, it is noteworthy that more environmental non-profit employees than scientists^11^ oppose shark fin bans, for essentially the same reasons given by scientists^14^. It is also noteworthy that support for fin trade bans is higher among those who never or rarely read the literature than among those who read it regularly.

## Conclusions

Surveyed scientists in^11^ raised concerns that some non-profit groups are not focusing on what scientific experts perceive as the most important problems, are not accurately describing the state of shark conservation threats, and are advocating for solutions not supported by the best available scientific data. Instances of those concerns were represented here primarily, but not exclusively, by employees of NGOs with certain shared characteristics: very small (less than 5 employees), based in North America or Europe, and employing advocates who report never reading the scientific literature or communicating with scientists. However, it should be noted that there are some very small NGOs who regularly work with scientists in support of science-based policies (e.g., Shark Advocates International), and it should be noted that some large NGOs who employ scientists have supported limit-based policies (e.g., the Pew Charitable Trusts with Shark Sanctuaries, and Oceana with shark fin trade bans), so size and interaction with scientists are clearly not the only predictors of policy preferences.

Typically (but not always), environmental policy change involves science and advocacy working together towards the same goal^16,24^ and therefore it is concerning to see stark differences in messaging between some high-profile shark advocacy campaigns and the policy recommendations of scientific experts—especially when some messaging uses demonstrably false information. Our results illustrate that despite notable instances of misinformation, many environmental non-profit employees work regularly with scientists, read the scientific literature, and support science-driven policy goals. This survey’s results also point to an opportunity to bring multiple forms of expertise [see^25^] and multiple perspectives to bear on conservation problems. Additionally, while scientists have previously identified scientifically inaccurate claims by advocates as a problem, advocates also point to meaningful, actionable ways scientists can increase the real-world relevance of their supposedly policy-relevant research: by engaging with the public, focusing on species and locations that are understudied, and committing long-term to research sites, local communities, and the development local scientific capacity. Advocates’ expertise and experience also includes and recognizes practical dimensions sometimes underrepresented in scientific research. For example, while it is a scientific fact that sustainable shark fisheries can and do exist and claims to the contrary are inaccurate, some advocates accurately point out that sustainable shark fisheries are uncommon, difficult to ensure, and present significant practical management challenges. While misrepresenting science is a problem, insights from stakeholders with diverse values, perspectives, and forms of expertise, including from those engaged in values-based approaches to advocacy, should be represented in discussions about shark conservation. The addition of these perspectives would make some conversations about conservation and management and associated trade-offs more nuanced and useful. This survey identified a communications problem in which some advocates may not have access to or be aware of certain scientific data; however, our results also highlight potential opportunities to more effectively address conservation problems through increased engagement between groups that too often talk past one another.

## Supplementary Information

Supplementary Information.

## Data Availability

In keeping with the terms of our human subjects research ethics permits, an anonymized version of the dataset used in this study with any possible identifying information removed is available upon request.
