# Supplementary material for: The role and value of science in shark conservation advocacy
Source: Sci Rep. 2021 Aug 17;11:16626. doi: 10.1038/s41598-021-96020-4 (PMC8370980; doi:10.1038/s41598-021-96020-4)
Supplement: Supplementary file 1 — Supplementary Information. [file 41598_2021_96020_MOESM1_ESM.docx]

**Supplementary Materials**

Table S1: A list of the environmental non-profit groups approached for this survey. For privacy reasons, we cannot report which non-profit groups responded to the survey.

| **Name** |
| --- |
| Abercrombie and Fish consultancy |
| Afri-Oceans Conservation Alliance |
| American Shark Conservancy |
| Animal Welfare Institute |
| Atlantic White shark conservancy |
| AZA SAFE |
| Bite Back |
| Bloom |
| Blue Ventures |
| Cape Eleuthera Institute (education arm) |
| Center for Biological Diversity |
| COARE |
| Defenders of Wildlife |
| EarthRace |
| Ecology Action Centre |
| Environmental Defense Fund |
| Environmental Justice Foundation |
| Fin Fighters |
| Fin Free |
| Fin Free Thailand |
| Fins and Fluke |
| Fins Attached |
| Flora and Fauna International |
| Gills Club |
| Greenpeace |
| Hong Kong Shark Foundation |
| Humane Society International |
| International Fund for Animal Welfare |
| International Seafood Sustainability Foundation |
| Keiko Conservation |
| LAMAVE |
| MarAlliance |
| Marine Conservation Science Institute |
| Marine Conservation Society of Australia |
| Marine Conservation Society UK |
| Marine Megafauna Foundation |
| Mission Blue |
| Monterrey Bay Aquarium (advocacy arm) |
| Mote Marine Lab (advocacy arm) |
| Nature Conservancy |
| New York Aquarium (advocacy arm) |
| Natural Resources Defense Council |
| Oceana |
| OceanCare |
| Pelagia Consultancy |
| PEW Environment Group |
| PRETOMA |
| Project AWARE Foundation |
| Sassama Consultancy |
| Save our Seas Foundation |
| Save Our Sharks Dutch Caribbean |
| Save Sharks Canada |
| Scottish Sea Angling Conservation Network |
| Sea Shepherd Conservation Society |
| Shark Advocates International |
| Shark Angels |
| Shark Defenders |
| Shark Friendly Marinas |
| Shark Research Institute |
| Shark Spotters |
| Shark Stewards |
| Shark Trust |
| SharkLab Malta |
| Sharks4Kids |
| SharkStuff |
| SharkTruth |
| Shedd Aquarium (advocacy arm) |
| South African shark conservancy |
| Support our sharks |
| Tennessee Aquarium (advocacy arm) |
| TRAFFIC |
| WildAid/Shark Savers |
| WildEarth Guardians |
| Wildlife Conservation Society |
| Wildlife Trust of India |
| WWF |
| Zoological Society of London |

Table S2
Survey questions, with the possible responses (MC= multiple choice, L= 1-5 Likert scale, or FR=free response) for each.

| **Question** | **Options** |
| --- | --- |
|  |  |
| *Demographics and personal background* |  |
| Please indicate your gender | MC |
| Please indicate your age | FR |
| Please indicate the highest level of education that you've obtained | MC |
| Are you a member of a scientific/professional society? | MC |
| If you are a member of a scientific/professional society, please indicate which one(s) | FR |
| Within your non-profit, what is your official title and (in your own words) your job description? | FR |
| What inspired your personal interest in shark conservation, in your own words (if applicable)? | FR |
|  |  |
| *NGO background* |  |
| Please indicate which of the following choices best describes the scope of your NGO's work (shark-focused, ocean focused, land and sea focused) | MC |
| Please indicate the geographic scope of your NGO's work (e.g., local/regional, national, international) | MC |
| If the geographic scope of your NGO is local/regional or national, please indicate the region or nation where your work is focused. | FR |
| Please indicate the approximate total number of paid employees of your NGO (not volunteers, not just the subset of your NGO working on shark conservation issues, all employees) | FR |
| Please indicate the 3 most common role that your NGO performs with respect to shark conservation (e.g., direct advocacy to decisionmakers for or against conservation policies, public education and outreach, providing funding to scientific researchers who perform conservation-relevant research, doing scientific research through a scientist employed by your organization, etc.) | FR |
| Are scientists involved in any capacity in your NGO? (E.g., scientists employed directly, formal scientific advisory board, etc). If so, how? | FR |
| What are up to 3 arguments that you or your NGO use to support the conservation of sharks, either in your advocacy or public education/outreach? | FR |
|  |  |
| *Interaction with science, scientists, and the scientific literature* |  |
| Have you personally worked with scientists, either independent scientists not employed by your NGO or scientists employed by your NGO? If so, please describe that experience in your own words | FR |
| Do you believe that most independent scientists not employed by your NGO are aware of and knowledgeable about current threats to sharks and current conservation efforts? Why or why not? | FR |
| Do you believe that science and scientists make a positive contribution to shark conservation efforts? Why or why not? | FR |
| If you could change one thing about how independent scientists not employed by your NGO interact with the conservation community, what would it be and why? | FR |
|  |  |
| If you could change one thing about the current focus of shark research, what would it be and why? | FR |
| In your opinion, what is the proper role of science and scientists in marine conservation? | FR |
| Do you personally believe that your NGO should incorporate peer-reviewed science into your proposed conservation solutions and advocacy? Why or why not? |  |
| Do you ever read the peer-reviewed primary scientific literature? If so, how often? Regularly (several papers per months), rarely (one paper per month or less), or never? | MC |
| If you do read the peer reviewed primary scientific literature, is it explicitly part of your job to do this? If you do not read the peer reviewed scientific literature, is the accessibility of papers a factor in that decision? | FR |
| Have you ever been a coauthor on a peer reviewed primary scientific literature paper? | MC |
| Which of the following options best reflects your opinion about the state of scientific research with respect to sustainable shark fisheries? The science is settled: sustainable shark fisheries can and do exist; the science is settled: sustainable shark fisheries cannot and do not exist; the science is uncertain. | MC |
| Why did you select the answer you did to the question above? | FR |
| If scientific research unequivocally showed that sustainable shark fisheries were possible, which of the following responses would best reflect your personal opinion? (If scientific research shows that sustainable shark fisheries are possible, I’d acknowledge that they are possible but be cautious about supporting them; If scientific research shows that sustainable shark fisheries are possible, I’d strongly support them; My opinion about shark fisheries is based on a belief that it is ethically or morally wrong to harvest sharks, so scientific measures of sustainability are not relevant to me) | MC |
| Several scientific papers have reported that the loss of sharks can cause ecosystem-wide negative effects (e.g., trophic cascades). Were you aware of these papers? Does this seem accurate to you? Do you use these results in your advocacy or outreach? | FR |
| Several scientific papers have reported severe and rapid shark population declines (e.g., 90% or more declines in the past few decades). Were you aware of these papers? Does this seem accurate to you? Do you use these results in your advocacy or outreach? | FR |
| Several scientific papers have challenged the idea of rapid and severe shark population declines and resulting ecosystem-wide negative effects, arguing that these papers are based on flawed data or flawed analysis of that data. Were you aware of these papers? Does this seem accurate to you? Do you use these results in your advocacy or outreach? | FR |
| Several scientific papers have shown that sustainable shark fisheries are theoretically possible, that sustainable shark fisheries exist, and that majorities of scientific researchers support sustainable fisheries over fishing and trade bans. Were you aware of these papers? Does this seem accurate to you? Do you use these results in your advocacy or outreach? | FR |
| Several scientific papers have reported population recoveries among once-overfished shark populations. Were you aware of these papers? Does this seem accurate to you? Do you use these results in your advocacy or outreach? | FR |
|  |  |
| *Shark conservation background* |  |
| In your opinion, what are the top threats facing shark populations overall? | FR |
| To the best of your knowledge, what are 3 species of sharks which are particularly threatened? | FR |
| In your opinion, are sustainable commercial fisheries that target sharks possible? | MC |
| Why do you believe (or not believe) that sustainable commercial fisheries that target sharks are possible? | FR |
| In your opinion, are there sustainable commercial fisheries targeting sharks that exist in the world today? | MC |
| If you believe that there are sustainable commercial fisheries targeting sharks in the world today, which fisheries are you referring to (species, location) | FR |
| In your opinion, should the general goal of shark conservation advocacy be to promote sustainable exploitation and trade of sharks and shark products, or to ban exploitation and trade of sharks and shark products? | MC |
| Why did you select the answer you did to the question above? | FR |
| Is there anything else you would like to say about the sustainability of shark fisheries? | FR |
|  |  |
| *Policy preferences* |  |
| What types of shark conservation policies have you and your NGO advocated for in the past 5 years? (Fishing restrictions with the goal of sustainable fisheries exploitation (e.g., fisheries quotas, RFMO agreements, required gear types, areas where fishing is allowed during certain times of year); Shark finning bans, which make it illegal to remove the fins of a shark at sea and discard the carcass at sea; International restrictions on shark trade (e.g., CITES Appendix I, CITES Appendix II, CMS); No-take Marine Protected Areas/Marine Reserves; Nationwide bans on shark fishing for any shark species (Shark Sanctuaries); Other) | MC |
| Please indicate the degree to which you feel the following shark conservation and management policy tools are appropriate and effective: (1 strongly disagree, 2 disagree, 3 neither agree nor disagree, 4 agree, 5 strongly agree) (This text is the same for each of the following 5 questions, indicated by …)  Fishing restrictions with the goal of sustainable fisheries exploitation (e.g., fisheries quotas, RFMO agreements, required gear types, areas where fishing is allowed during certain times of year) | L |
| …Shark finning bans, which make it illegal to remove the fins of a shark at sea and discard the carcass at sea | L |
| … International restrictions on shark trade (E.g., CITES Appendix I, CITES Appendix II) | L |
| …No-take Marine Protected Areas/Marine Reserves | L |
| …Nationwide bans on shark fishing for any shark species (Shark Sanctuaries) | L |
| …Shark fin bans which make it illegal to buy, sell, or possess shark fins | L |
| Is there anything else you would like to say about any of these policies? | FR |

Table S3: Representative examples of how certain environmental non-profit groups discuss shark conservation threats, solutions, and reasons to support shark conservation in general from websites and press releases.

| **Threat** | **NGO** |
| --- | --- |
| “Despite their image, sharks are among the most valuable, vulnerable, and neglected creatures in the ocean. Over hundreds of millions of years, sharks have evolved to serve as important ocean predators and, as such, are not well equipped to withstand heavy predation themselves. In fact, most sharks are exceptionally susceptible to overfishing due to slow growth, late maturity, and small numbers of young.” | Shark Advocates International |
| “Some species have fallen by 90% over this period and, today, approximately one-third of all shark species are already listed as threatened with extinction, now or in the near future (IUCN  Red List 2012). Critically, this situation is one that directly impacts the health of our oceans: As sharks are apex predators, they sit at the top of the marine food chain and help to regulate the abundance and trophic diversity of the different species beneath them. | Hong Kong Shark Foundation |
| “Shark fin soup is cruel and wasteful. Fins removed, the animals are thrown back into the water to die slowly and painfully.” | Humane Society International |
| “most sharks caught as bycatch in Canada either have no limits placed upon catches, or have catch limits that are far too high or unenforceable, and therefore have little conservation value. There is also a lack of scientific knowledge about most of these shark species – for many, we don’t have a good grasp of how healthy their populations are, where and when they reproduce or how many we’re actually catching. This makes it really hard to make educated, science-based decisions on how many can be sustainably caught, and unfortunately Canadian fisheries management has not been erring on the side of caution.” | Ecology Action Centre |
| “Great white sharks are a critical part of the ocean ecosystem, playing an important top-down role in structuring the ecosystem by keeping prey populations in check, like sea lions and elephant seals. The presence of great white sharks ultimately increases species’ stability and the diversity of the overall ecosystem” | Center for Biological diversity |
| “These majestic top predators that are so essential to the natural order of marine ecosystems now face their most severe threat from overfishing. Many species are threatened with extinction | WWF |
| “Sharks have been swimming in the oceans since before dinosaurs walked the earth. They’ve shaped the marine environment and everything in it. Without sharks the oceans could collapse, taking with them their ability to produce 50% of the oxygen we breathe and absorb 20% of the CO2 emissions we produce.” | BiteBack |
| “Because they grow slowly and produce few young, sharks are exceptionally susceptible to overexploitation. Further, a healthy and abundant ocean and the communities that rely on it depend on healthy shark populations. Living sharks can help fuel local economies in places like Palau, where sharks bring in an estimated $18 million per year through dive tourism.” | Project Aware |
| Worldwide, at least 100 million sharks are killed each year in commercial fisheries; nearly 30 percent of all known shark species assessed by scientists are threatened with extinction. Sharks play an important role in maintaining the health of the entire ocean, but they grow and reproduce slowly, which makes them particularly vulnerable to overfishing. Officials in many coastal and island nations recognize sharks’ high value to the dive tourism industry.  “Establishing sanctuaries to protect all sharks makes clear that these top predators warrant the same status as other vulnerable marine wildlife that help attract ecotourism, such as turtles and whales,” Warwick added. | PEW environment group |

TABLE S4 How policy solutions are discussed on NGO websites.

| **Solution** | **NGO** |
| --- | --- |
| “focused on publicizing the plight of sharks and advocating science-based policies on their behalf before fishery management and wildlife conservation bodies” | Shark Advocates International |
| “Hong Kong has the power. Over 50% of the world’s shark fin is traded through Kong Kong. If the people of Hong Kong take a stand against shark fin, the whole world will listen.” | Hong Kong Shark Foundation |
| “The future of sharks and rays – and the health of our ocean planet – hinges on keeping fishing and trade at sustainable levels.” | Project AWARE |
| “a nationwide ban on the trade of shark fins would reduce the international fin trade, improve enforcement of the current finning ban, and reinforce the status of the United States as a leader in shark conservation.” | OCEANA |
| “The EAC fully supports measures that reduce the number of sharks that are killed unsustainably and wastefully.” | Ecology Action Centre |
| “AWI is leading an effort to compel restaurants in the US that currently serve shark fin soup to cease doing so because of the cruelty of shark finning and the fragility of shark populations.” | Animal Welfare Institute |
| “our aim is to stop the over-exploitation of sharks in at least 40 priority countries. we aim to put in place effective trade controls. This will ensure international trade in shark products is legal, sustainable and traceable. | Shark Trust UK |
| “AMCS does not currently support any targeted shark fishing in Australia, and recommends that the public avoids shark or ‘flake’ when eating seafood | Australian Marine Conservation Society |
| “For coastal developing countries, fishing for a range of species is often important for the livelihood, culture, and health of the human population. However, in many regions, catches are diminishing and the sizes of captured fishes is decreasing. Collecting data from fisheries can allow researchers to identify and address problems to ensure the continued health of fish populations and the communities that rely upon them.” | MarAlliance |
| “Completing the NPOA (National Plan of Action) is a pivotal step for shark conservation in Cuba and provides a clear policy mandate for future regulatory and management actions taken to protect sharks. | EDF |
| “An Endangered Species Act listing will afford the sharks protections from key threats and garner funding for research to better understand the status and threats to this distinctive population of white sharks. | Center for Biological Diversity |
